# 

**Published:** 1883-12

**Authors:** 


### INDEX TO VOLUME XLVII.

### A.

PAGE.

Abcess of the Tongue......... 175
Abdominal Section for Intestinal Obstruction, Parkes. ... 372
Abstracts, Ryerson............ 556
Accidents and Emergencies, Dulles, Review............. 488
A Doctor's Sanctum .......... 670
Anæsthetic Mixture, Byrd...... 283
After-Treatment of Puerperal Women, Keating.......... 306
Axillary Hyperidrosis ........ 389
Albumenuric Retinitis of Pregnancy .................. 556
Alumni Association, Chicago Medical College.... .. .... 103
American Medical Association 51
American Surgical Association 151
American Dermatological Association, Announcement ..... 288
American Microscopical Association, Item............... 336
American Academy of Medicine, Announcement.........420
American Gynæcological Society, Review ............ .. 486
Amennorrhoea in Morphine Eaters .......... ... ... 177
Animal Heat and its Equivalent, Valin .. ............ 241
Anguillula.................. 173
Antiseptic Catgut............. 405
Antiseptic Remedies in Diseases of the Eye and Ear, Holmes 225
A Royal Nurse............... 112
Asylums of Great Gritain, Brush 494
Atlee, John M., Opening Address at American Medical Association, June 4, 1883.... 1
Atropine in Epilepsy ........ 176
Bacratosis.................. 181

### B.

PAGE.

Bartlett, E. A., Electricity in Uterine Displacements...... 208
Becker, W. F., Prof. Draper's Clinics.................... 384
Belfield, W. T., Notice........ 108
Bennett, S. F., Prolonged Gestation.................... 130
Bennett, H. J., Seasickness and its Prevention.... ......... 42
Berlin Polyclinic............. 50
Books and Pamphlets Received ..................167, 488, 666
Bothriocephalus.............. 176
Bradley, W. H., Item......... 240
Bright's Disease, Treatment, Purdy ..................... 561
Brower, D. R., Concealed Insanity.... ......... . ..... 289
Brush, N. E., Asylums of Great Britain ....... ........... 495
Burdick, P. D., Postpartum Malarial Fever ................ 254
Byford, W. H., Intra-Pelvic Inflammation in the Chronic Form .... ............... 421
Byford, W. H., Jr., Obituary .. 555
Byrd, Wm., a Letter from..... 283

### C.

Calomel, Influence on Digestion 543
Cannabis Indiea ............. 544
Card to the Public............ 480
Caries, Treatment of.......... 189
Carter, J. M. G., Correction of Former Article........... . 554
Carter, J. M. G.,Cerebral Hyperæmia .................... 362
Chicago Medical Society...... 159
Meeting May 21, 1883...... 93

PAGE.

Meeting May 7, 1883........ 86
Meeting June 18, 1883 ...... 262
Meeting July 2, 1883 ... ... 269
Meeting July 16............ 390
Special Meeting, Oct. 26..... 481
Chicago Pathological Society, Annual Meeting ........... 97
Chicago Pathological Society, Annual Report of........... 101
Meeting, June 11, 1883...... 272
Meeting, July 9............ 276
Meeting, Aug. 13, 1883...... 395
Meeting, Sept. 10, 1883...... 398
Meeting, Oct. 8, 1883........ 627
Chicago Throat and Chest Hospital ...................... 671
Correspondence, Domestic.... ....... .......... 401, 555, 616
Craig, A. L., Patent Medicine.. 41
Credit to whom Credit is Due. 220
*Centralblatt*, Confiscation of .. 112
Cerebral Hyperæmia, Carter .. 362
Cholera Scare, Reilly ......... 477
Clevenger, S. V., Insanity in Chicago .......... ....... 449
Co-Existence of Diptheria and Typhoid Fever, Paget, C. E.. 328
Concealed Insanity, Brower... 289
Cholecystotomy .............. 189
Chlorate of Potash Poisoning.. 187
Cincinnati Board of Health, Item...................... 280
Clevenger, S. V., Notice....... 109
Cook County Hospital, Item... 108
Compression of the Ovary..... 171
Convallaria ................. 171
Convulsions in Children ...... 138
Cotoine .................... 169
Croupous Pneumonia, Etiology of ....................... 413
Coulson on the Bladder and Prostate Gland, Review ..... 411
Cobweb in Intermittent Fever. 419
Cold Pack and Massage Treatment of Anæmia, Jacobi and White, Review ...... ..... 487
Correction, Carter, J. M. G. ... 554
Cushing, G. H., Pre-Natal Influence on Teeth............ 610

D.

Delirium Grave, Post-Mortem of ....................... 547
Deflection of the Septum Narium, Ingals ............... 128
Diabetes Treated with Codeia, Bradbury, Dr,............ 440

PAGE.

Diptheria, Keating .......... 519
Diseases of Children, Palatable Doses in, Churchill......... 659
Diseases of the Ear in Children, Von Topvaltsch, Review....... .............. 287
Diseases of Women, Hewitt, Review .................. 411
Diseases of Women, Edis Review...................... 485
Durhæmatoma.... ........... 131
Dyas, W. G., Lymphatic System 337

E.

Easton, C. M., Letter from..... 282
Ecstasy ..................... 224
Edis, A. M.,Diseases of Women, Review .................... 485
Editorial.............106, 490, 640
Eggemann, C. R., Exterge Oculum ...................... 252
Electricity in Uterine Displacements, Bartlett ............. 208
Electricity in Intermittent Fever ........................ 412
Fllis, C, B., Letter from....... 284
Empyema, Treatment of, Ingals ...................... 113
Encephalic Hemorrhage...... 174
Ergot in Locomotor Ataxy.... 314
Errata ...................... 223
Essentials of Pathology, Gilliam, Review .............. 410
Etheridge, J. H., Removal of Both Ovaries............... 11
Excision of the Hip Joint, Owens .................... 378
Explanation. ................ 645
Exterge Oculum, Eggemann.. 252

F.

Fibroma of Kidney........... 183
First aid to the Injured Shepherd, Review............... 164
Foreign Body in the Urethra.. 557
Frequent Repetition of Doses, Martin..................... 655
Foreign bodies in the Ear, Hotz....................... 20
Foreign Body in the Trachea.. 282

G.

Gastronomy. ................ 177
Germ Theory of Typhoid Fever and Treatment of this Disease by Salicylate of Bismuth.... 322

PAGE.

Gihon, A. L., Address on State Medicine .................. 191
Goding, Human Parasites..... 587
Gordon, C. A., Cholera and Its Treatment.................. 317
Gonorrhœal Sciatica.......... 224
Green, T. H., The Tubercle Bacillus and Phthisis .......... 647
Gynecology of the Ancients. Jenks. Review ............ 406

H.

Haemetic Origin of Bright's Disease .................... 179
Hay Fever Treatment, Phillip, W. F.......................... 330
Health, State Board. Letter from Secretary............. 401
Health, Illinois Board of...... 560
Hospital Clinics Report by W. F. Becker.................. 384
Hospital Reports, by Drs. Davis and Currie ................. 259
Holmes, E. H., Antiseptic Remedies.......................
Homœopath's Faith, Jones, Review....................... 487
Homœopathy in Vienna....... 278
Hotz, F. C., Foreign Bodies in the Ear .................... 20
Hyde, James Nevins Notice... 223
Same, Item .................. 288
Hygienic Treatment of Albubuminuria, Shattuck, G. B... 335
Hypodermic Injections of Ether in Pneumonia.............. 646
Hyperosmic Acid Injection of Tumors ..................... 185

I.

Icterus Neonatorum.......... 176
Imperforate Anus............. 177
Ingals, E. Fletcher, Treatment of Empyema.................. 113
Inhalation of Oxygen in Gas Poisoning, Alonzo Clark.... 657
Inheritance of Cancer......... 216
Insane Asylum, Visit to ...... 10
Insanity in Chicago, Clevenger 449
Intra-Uterine Injections in Puerperal Septicœmia, Thomas .. 213
Intra-Pelvic Inflammation in the Chronic Form, Byford... 421
Iodoform in Lung Affections .. 418
Items............ 109, 221, 559, 667

PAGE.

J.

Jenks, E. W., Item............ 258
Jequirity Ophthalmia ....... ... 170
Jones, S. J., Item............ 288
Journal, Item ............. ... 288

K.

Keating, J. M., Diptheria...... 519
After Treatment of Puerperal Women.................... 306
King, O. S., Item............. 336
Knox, J. S., Item ............. 336

L.

Lactic Acid in Diphtheria, Lytle ...................... 388
Lactic Acid, Apology for the Printer's Errors........... 412
Lady Doctors in China, Item.. 280
Lead Palsy.................. 184
Legal Medicine Tidy, Review.. 408
Leonard, R. L., Item.......... 240
Letter from Cincinnati........ 616
Library of Surgeon General's Office, Item ................ 285
Lydston, G. F., Treatment of Varicocele ................. 351
Lyman, H. M., Potatoes and Tobacco .................... 473
Lytle J. P..................... 388
Lymphatic System, Dyas...... 337

M.

Mac Cormac, Sir William, Reception to.................. 481
Macalina ..................... 405
Malignant Thyroid Tumors.... 174
Manheimer, M., Typhoid Fever 124
Marine Hospital Service ...... 669
Marriages in Paris ........... 190
Martin, J. W., Repetition of Doses...................... 655
Martin, F. H., Massage, its Application..................... 26
New Operating Table......... 34
Massage, Its Application, Martin......................... 26
Massage ..................... 558
Medical Frauds............... 221
Menstruation in the Typhoid Condition ................. 188
Mercurial Intoxication........ 150
Mercuric Bi-Chloride Antiseptic Method, Kuemmell's........ 445
Merriman, H. P., Notice ...... 108

PAGE.

Microbes in Fishes ........... 186
Micro-Organisms of Disease, Belfield, Review ............... 286
Michigan State Board of Health 629
Miller DeLaskie, Notice ....... 108
Milk, Effects of Medicine on .. 190
Miller DeLaskie, Item.... ... 240
Minnesota College Hospital.... 555
Moluscum Simplex........... 622
Moral Insanity, Workman..... 531
Mortality for May, 1883 ....... 50
For September.............. 615
Myxœdma, Robinson .......... 467

N.

Naso-Pharyngeal Tumor, Case of, Olney................. 48
National Conference of Charities, Report of.............. 631
Neuralgia, Treatment of...... 299
New Operating Table, J. N. Martin.......... .. ....... 34
Nitrate of Amyl and Nitro-Glycerine, in Uræmsc Asthma, Dr. Sheen................... 331

O.

Obituary.................555, 663
Obstetrical and Gynecological Society of Washington, D. C. 200
Ogden, Henry, Triplets........ 228
Olney, J. E., Naso-Pharyngeal Tumor ....... ............. 48
Opening Address, J. W. Atlee. 1
Opium, The True Antidote for, Stearns .................... 47
Selections.................... 413
Society Reports .............. 390
Original Communications, 11, 113, 225, 449, 337, 561
Original Translations......... 138
Out of Fashion Remedy, Hare, C. J....................... 438
Ovaries, Diseases of, Tait... . 163
Owens, J. E., Excision of Hip Joint ...................... 378

P.

Paramyo Klonus Multiplex.... 173
Parasites, J. W. Goding ....... 587
Park, Roswell, Item .......... 224
Parkes, C. T., Exploratory Section of the Abdomen........ 372

PAGE.

Park, Roswell, Removal....... 137
Patent Medicine, Craig........ 41
Patterson, R. J., Item ........ 336
Pay Hospitals, Review........ 105
Phenic Acid.................. 186
Pharmacopœia of 1880, Corrections in .................... 666
Picric Acid Test for Albumen and Sugar in the Urine...... 217
Picric Acid Test for Albumen, Johnson, Geo............... 333
Porro's Operation ......... ... 137
Pospartum Malarial Fever, Burdick ............ .......... 254
Postepileptic Phenomena, Althaus, Julius .............. 436
Potatoes and Tobacco, Lyman, H. M. ................. .... 473
Potts' Disease, Shafter, Review. 165
Practice of Medicine, Bartholow, Review................ 409
Practitioners' Courses, Item ... 108
Pre-Natal Influence on Teeth, Cushing ................... 610
Presentation of Child's Head in Vulva and Anus ............ 672
Prolapsus of the Rectum....... 279
Prolonged Gestation, S. F. Bennett.................... .. 130
Ptyalism in the Insane........ 188
Purdy, Chas. W., Treatment of Bright's Disease ............ 561

R.

Rhea, Frank L., Notice ..... 420
Reviews and Book Notices .... 420
Reily, F. W., Cholera Scare ... 477
Resection of the Stomach ..... 416
Resorcin in Diphtheria........ 420
Reynolds, H. J., Etiology of Urethral Inflammation ......... 36
Removal of both Ovaries, Etheridge.................... ...... 11
Reparative Surgery of the Genital Tract, Pallen, M. A...... 326
Resorcin ...................... 414
Reynolds, T. U., Timely Catharsis ........................ 132
Rhea, Frank L., Obituary by R. L. Rhea .................. 663
Rheumatic Ophthalmia....... 189
Robinson, J. A., Mxyœdema... 467
Ross, J. P., Item.............. 336
Ryerson, Dr., Albuminuric Retinitis of Pregnancy......... 556

PAGE.

S.

Scott, D., Serpiginous Phagedena ........ 465
Sea-Sickness and Its Prevention, Bennett, H. J. ........ 442
Skin Diseases ........ 219
Cmith, Chas. Gilman, Item .... 224
Society Meetings, Oct. 15, 1883. 623
Spinal Curvature ........ 545
State Medicine, Address on, Gihon ........ 191
Stearns, J. H., The True Antidote for Opium ........ 47
Stockton, Frank O., Item ........ 662
St. Louis Medical Society and Code of Ethics ........ 448
Sternum, Resection of ........ 389
*St. Louis Medical and Surgical Journal*, Changes in Staff .... 639
Strausser, Simeon, Item ........ 336
Sulpho-Tartrate of Quinine ... 417
Syphilitic Indigestion ........ 108

T.

Tait, Lawson, Diseases of the Ovaries ........ 163
The Living Death ........ 667
Therapeutics of the Skin, Piffard, Review ........ 407
Thometz, Dr., Typhoid Fever .. 124

PAGE.

Thomas, T. G., Intra Uterine Injections ........ 213
Thomsen's Disease ........ 173
Timely Catharsis, Reynolds .... 132
Translations from Foreign Exchanges ........ 169, 279, 413
Triplets, Ogden ........ 228
Tri-State Medical Society ...... 162
Tubercle Bacillus and Phthisis, Green ........ 647
Typhoid Fever, Manheimer and Thometz ........ 124

U.

Urethral Inflammation, Etiology of, Reynolds ........ 36
Urethro Rectal Fistula ........ 175

V.

Valin, H. D., Animal Heat .... 241
Varicocele, Treatment of, Henry Review ........ 166
Varicocele, Lydston ........ 351

W.

Wagner, the Vegetarian ........ 112
Wakeman, E. L., Announcement ........ 559
Wells, Sir T. Spencer ........ 182
Wilbur, Henry B ........ 220
Workman, Moral Insanity ..... 531

### BOOKS RECEIVED.

Lindsay & Blakeston's Visiting List for 1884. Twenty-five patients, price $1; fifty patients, price $1.25.

Physicians' Memorandum Book and Weekly Visiting List. Thirty-one to sixty-two patients, $1.25.

A Manual of Psychological Medicine and Allied Nervous Diseases. Octavo, 699 pp. Price $5. P. Blakiston, Son & Co.

The Physiological Factor in Diagnosis. I. Milner Fothergill. Wm. E. Wood & Co., through W. T. Keener, 96 Washington St., Chicago.

---

ANY person having purchased a copy of the United States Pharmacopœia of 1880, and desiring a list of the corrections since made therein, can procure same by sending a two-cent stamp to

WM. WOOD & CO., Publishers,
56 and 58 Lafayette Place, New York.

